# SARS-CoV-2 infection and immune responses

**DOI:** 10.3934/microbiol.2023015

**Published:** 2023-03-29

**Authors:** Rakhi Harne, Brittany Williams, Hazem F. M. Abdelaal, Susan L. Baldwin, Rhea N. Coler

**Affiliations:** 1 Seattle Children's Research Institute, Center for Global Infectious Disease Research, Seattle Children's Hospital, Seattle, Washington, USA; 2 Department of Global Health, University of Washington, Seattle, Washington, USA; 3 Department of Pediatrics, University of Washington School of Medicine, Seattle, Washington, USA

**Keywords:** SARS-CoV-2, COVID-19, immunopathology, vaccines, antibodies, inflammation

## Abstract

The recent pandemic caused by the SARS-CoV-2 virus continues to be an enormous global challenge faced by the healthcare sector. Availability of new vaccines and drugs targeting SARS-CoV-2 and sequelae of COVID-19 has given the world hope in ending the pandemic. However, the emergence of mutations in the SARS-CoV-2 viral genome every couple of months in different parts of world is a persistent danger to public health. Currently there is no single treatment to eradicate the risk of COVID-19. The widespread transmission of SARS-CoV-2 due to the Omicron variant necessitates continued work on the development and implementation of effective vaccines. Moreover, there is evidence that mutations in the receptor domain of the SARS-CoV-2 spike glycoprotein led to the decrease in current vaccine efficacy by escaping antibody recognition. Therefore, it is essential to actively identify the mechanisms by which SARS-CoV-2 evades the host immune system, study the long-lasting effects of COVID-19 and develop therapeutics targeting SARS-CoV-2 infections in humans and preclinical models. In this review, we describe the pathogenic mechanisms of SARS-CoV-2 infection as well as the innate and adaptive host immune responses to infection. We address the ongoing need to develop effective vaccines that provide protection against different variants of SARS-CoV-2, as well as validated endpoint assays to evaluate the immunogenicity of vaccines in the pipeline, medications, anti-viral drug therapies and public health measures, that will be required to successfully end the COVID-19 pandemic.

## 1. Introduction

Humanity has suffered since ancient times from infectious diseases in epidemic or pandemic situations causing public health emergencies [1],[2]. Emerging viral pathogens pose a great threat to the community, particularly due to the naïve immune state of the individual and in circumstances of high human-to-human transmission via inhalation, or other means of infection. Severe acute respiratory syndrome coronavirus 2 (SARS-CoV-2) is the causative agent of coronavirus disease 2019 (or COVID-19) that targets the lower respiratory tract in humans. COVID-19 was identified in December 2019 from the city of Wuhan in Hubei province when cases emerged in humans, likely, but not confirmed, from a zoonotic transmission in China[3]–[7]. SARS-CoV-2 has rapidly spread to other parts of the world due to its ability to transmit from human-to-human [8]. SARS-CoV-2 was declared a pandemic by the World Health Organization (WHO) on March 11^th^, 2020, when the number of cases increased exponentially across multiple countries in the world. As of February 21^st^, 2023, according to the WHO, there have been 757,264,511 confirmed cases of COVID-19 and 6,850,594 deaths globally [9] and approximately 13.2 billion vaccine doses have been administered. Over 1 million US deaths have occurred as per the data from the Centers for Disease Control and Prevention (CDC) [10].

All age groups are susceptible to SARS-CoV-2, however infected individuals exhibit a range of clinical severity. COVID-19 patients can be asymptomatic, symptomatic, experience mild symptoms, or can have severe illness leading to death. Whereas most COVID-19 patients exhibit mild symptoms, older adults, and certain people with underlying medical conditions, such as cardio-vascular and metabolic abnormalities, can become severely ill and are at higher risk of infection [11],[12]. SARS-CoV-2 primarily enters the host via the nasal mucosa and targets the alveolar type II epithelial cells lining the respiratory tract in lungs causing an infection in the upper airway epithelium [13]. Symptoms usually appear 2-14 days after exposure to the virus and can include fever or chills, cough, shortness of breath or difficulty in breathing, fatigue, muscle or body aches, headache, new loss of taste or smell, sore throat, congestion or runny nose, nausea or vomiting, and/or diarrhea [14],[15]. SARS-CoV-2 infection induces a cytokine storm in critical COVID-19 patients [16]–[19]. Cytokine storm is a condition in which cytokines are produced excessively by a dysfunctional immune system causing systemic hyperinflammation, acute respiratory distress syndrome (ARDS) and multi-organ damage which ultimately leads to physiological deterioration and death. In a study examining tracheal aspirates from COVID-19 associated ARDS patients with the ARDS induced from other pathogens, it was observed that the immunological features of COVID-19 associated ARDS are unique [20],[21]. With COVID-19 induced ARDS, there is reduced expression of pro-inflammatory genes and upregulation of genes involved in non-canonical roles in inflammation, immunity and interferon signaling [20]. COVID-19 primarily affects the respiratory system including lungs [11],[22]–[30], however pathogenesis in the cardio-vascular system [31]–[33], neuromuscular system [34], nervous system[35]–[38], and associated with metabolic dysfunctions [12],[39],[40] and Diabetes [41],[42] have also been identified [43].

Coronaviruses (CoVs) are RNA viruses that belong to the *Coronaviridae* family and genus *Betacoronavirus* [44]. SARS-CoV-2 characterized as a member of *Betacoronavirus* genus is a single-stranded ribonucleic acid (ssRNA) virus enveloped in a lipid membrane [45]. SARS-CoV-2 virus was discovered to be closely related to the CoVs found predominantly in bats (88% identity) and are sufficiently distant from other CoVs including Severe Acute respiratory syndrome coronavirus (SARS-CoV) (79% identity) and Middle East respiratory syndrome (MERS) coronavirus (50% identity) [6],[46]. CoVs cause a variety of diseases targeting the respiratory system from mild colds to more severe diseases. CoVs have caused epidemic disease in humans such as SARS in 2003[47] and MERS in 2012 [48].

**Figure 1.**
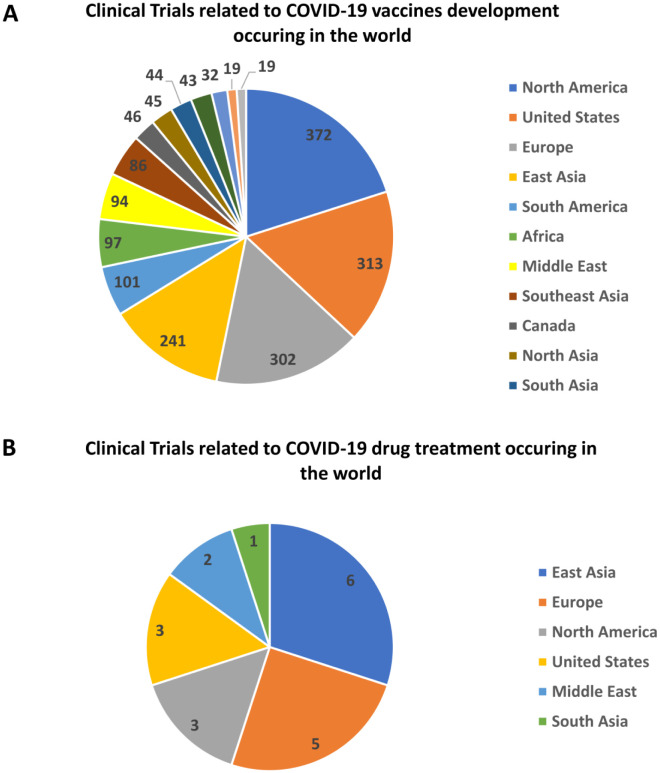
Number of Clinical Trial studies occurring across the world depicting widespread research on COVID-19 therapeutics as posted on ClinicalTrials.gov (23^rd^ February 2023). Ongoing clinical trials related to COVID-19 vaccine development (A) and COVID-19 drug treatment (B) across regions. Search terms included COVID-19 vaccine and COVID-19 drug treatment respectively.

Almost three years since the pandemic, there has been increased interest in studying how the immune system combats SARS-CoV-2 infections. Since February 2023, over 8000 clinical trial studies have taken place worldwide; many of which include drugs and vaccines in different stages of testing against COVID-19. Around 1400 of these studies are on vaccine development and 193 are focused on COVID-19 drug treatment in different clinical phases. A summary of COVID-19 related clinical studies and the regions in which they are occurring are shown in Table 1 and Figure 1 (ClinicalTrials.gov). With ongoing cases of COVID-19, caused by continuous mutations and evolving variants, additional solutions and resources are needed to enhance the available vaccine and drug treatment pipeline. Furthermore, more effort and resources are required to identify immunological correlates of infection and markers of protection, to help facilitate development of diagnostic tests, effective vaccines and therapeutic antibodies.

**Table 1.** Summary of the Clinical Studies related to COVID-19 (Reported in ClinicalTrials.gov, 21^st^ Feb 2023).

Clinical Phase	Total number of COVID-19 clinical studies	COVID-19 vaccine related clinical studies	COVID-19 drug treatment related clinical studies
Early phase 1	65	12	2
Phase 1	692	246	30
Phase 2	1538	322	62
Phase 3	978	240	49
Phase 4	266	99	11
Not applicable	2041	198	23

## 2. SARS-CoV-2 variants

SARS-CoV-2 variants are designated by the WHO as Variants of concern (VOC) or Variants of interest (VOI) depending upon their risk of infection and transmission [49]. VOC cause widespread numbers of cases globally with increased virulence due in part to the reduced effectiveness of available vaccines and therapeutics, as well as acquired immunity from prior infection with a different variant. VOI cause significant community transmission in multiple countries but is not known to become widespread. Currently, there are no circulating SARS-CoV-2 VOIs, however Omicron and its sub-variants (from B.1.1.529 to XBB.1.5) have become VOC, causing a wave of COVID-19 community transmission leading to worldwide deaths since its discovery in South Africa in November 2021 [49]–[51]. In the United States, the CDC classifies SARS-CoV-2 variants in another category called Variants being monitored (VBM) which includes variants previously associated with more severe disease cases and increased transmission but are no longer a threat to the public health. A list of SARS-CoV-2 VOC, VOI and VBM based on their date of emergence and category classification is shown in Table 2. Genomic surveillance, high sequencing volumes and quick turnaround times allow early detection of VOC. VOC were identified as Alpha (B.1.1.7), Beta (B.1.351), Gamma (B.1.1.28) (P.1), Delta (B.1.617.2) and Omicron (B.1.1.529) variants [52].

**Table 2.** SARS-CoV-2 variants classified as VOC, VOI and VBM based on emergence date (Source: WHO and CDC [49],[53]).

WHO Label	Pango Lineage	Earliest documented samples	Date of Designation
Alpha	B.1.1.7 and Q lineages	United Kingdom, Sep-2020 [49],[54]	VOC, December 29, 2020		VBM,September 21, 2021
Beta	B.1.351 and descendent lineages	South Africa, May-2020 [49],[55]	VOC, December 29, 2020		VBM, September 21, 2021
Gamma	P.1and descendent lineages	Japan/Brazil, Nov-2020 [49],[56]	VOC, December 29, 2020		VBM, September 21, 2021
Delta	B.1.617.2 and AY lineages	India, Oct-2020 [49],[57]	VOC, June 15, 2021		VBM, April 14, 2022
Epsilon	B.1.427	California, December 2020 [58]	VOC, March 19, 2021	VOI, February 26, 2021	VBM, September 21, 2021
Epsilon	B.1.429	California, December 2020 [58]		VOI, June 29, 2021	
Eta	B.1.525	United Kingdom/Nigeria, December 2020 [58]		VOI, February 26, 2021	VBM, September 21, 2021
Iota	B.1.526	New York, USA, November 2020 [49],[59]		VOI, February 26, 2021	VBM, September 21, 2021
Kappa	B.1.617.1	India, December 2020 [58]		VOI, May 7, 2021	VBM, September 21, 2021
N/A	B.1.617.3	India, December 2020 [58]		VOI, May 7, 2021	VBM, September 21, 2021
Zeta	P.2	Brazil, April 2020 [49]		VOI, February 26, 2021	VBM, September 21, 2021
Mu	B.1.621, B.1.621.1	Colombia, January 2021 [49],[60]			VBM, September 21, 2021
Omicron	B.1.1.529, BA.1, BA.1.1, BA.2, BA.3, BA.4 and BA.5, XBB, XBB.1, XBB.1.5	Multiple countries, Nov-2021 [49],[61]	VOC, November 26, 2021		

The Alpha variant which was first recognised in September 2020 in the United Kingdom (UK), became a dominant strain in the UK in the first half of 2021 [49],[54]. The N501Y mutation, found in the Receptor Binding Domain (RBD) region of the spike protein of the Alpha strain, [62]–[65] is known to have a greater affinity for the human angiotensin-converting enzyme 2 (ACE2) receptor than wild type RBD. This interaction increases viral adhesion, entry into the host cells and higher viral load leading to the greater transmissibility of the Alpha Strain [62],[65]. The N501Y mutation is also found in the RBD region of the Beta variant. In cohort studies held in England from late 2020 to early 2021, it was identified that the Alpha variant increased the risk of mortality between 61%–64% during COVID-19 infections [64],[66]. Earlier in May 2020 the first cases of the Beta strain were identified in South Africa, which brought about a second wave, however the proportion of both the Alpha and Beta strain transmission cases have continued decreasing since April 2021. It was observed that the three mutations in the RBD region of the Beta strain K417N, E484K and N501Y substitutions, did not increase infectivity but may have led to immune invasion from neutralising antibodies [67]–[69]. An investigational vaccine against the Beta strain mRNA-1273.351 from Moderna is being evaluated currently for safety and immunogenicity in adult volunteers by the National Institute of Allergy and Infectious Diseases (NIAID) (NCT04785144).

The Gamma (B.1.1.28) (P.1) variant was found in Brazil in November 2020 [55]. By April 2021 it was rapidly transmitted in 36 countries including the United States, Canada and Belgium along with the local transmission in Brazil [70]. In a study analysing epidemiology modelling of genomic and mortality data, it was demonstrated that the Gamma strain may be 1.7- to 2.4 times more transmissible than the Alpha and Beta strain, and it is relatively resistant to the neutralizing antibodies acquired from both convalescent plasma and vaccination [71],[72]. The Delta variant was originally identified in India in October 2020 [49],[59],[73] but rapidly became a dominant virus strain globally causing a new wave of SARS-CoV-2 infections and caused a second wave of SARS-CoV-2 infections in India. The Delta variant is the most infectious variant to date with the highest transmissibility compared to the Alpha, Beta and Gamma variant strain. In a study examining local transmission of the Delta strain in China, it was demonstrated that individuals infected with Delta variant had 1000 times more viral load than the Wuhan SARS-CoV-2 virus implying higher viral replication during Delta infections [57]. Another study which examined interactions between the Delta strain RBD mutations with the ACE2 receptor and neutralizing antibodies showed reduced interactions between RBD and neutralizing antibodies leading to immune evasion[74]. This is probably why there was rapid transmission of the Delta strain worldwide, until the Omicron variant became the predominant global variant beginning in November 2021 [49],[61].

**Figure 2.**
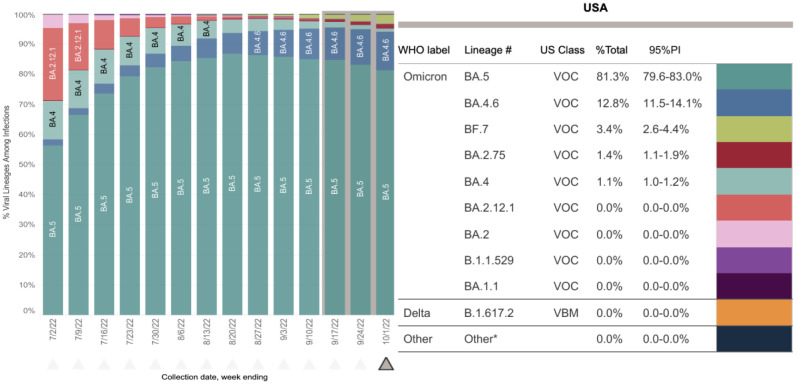
Genomic surveillance of the proportions of circulating Omicron sub-variants in the United States (07/02/2022 to 10/01/2022). Lineages are VOC sub-variants of Omicron or Delta strain in USA and circulating above 1% nationally in at least one week period. *Other represents the aggregation of lineages which are circulating <1% nationally during all weeks displayed[81].

The current VOC Omicron strain, first detected in South Africa, can quickly infect recovered individuals as well as vaccinated individuals [75],[76]. The Omicron variant has around 32 mutations in the spike glycoprotein, 15 of which are present in the RBD. In a study which tested 51,281 people positive either for Omicron or Delta variant, it was estimated that there was a 48% higher risk of viral transmission with the Omicron strain compared to the earlier Delta strain [51]. Several sub-lineages of the Omicron variant such as B.1.1.529, BA.1, BA.1.1, BA.2, BA.3, BA.4 and BA.5 have been identified [49],[77]. The substantial number of mutations in the spike protein has also been associated with reduced vaccine-induced neutralizing antibody responses [75],[77]–[79]. Based on the weekly update released by the WHO on 28^th^ September 2022, persistent increases in cases with diversity within the Omicron strain and its descendent lineages are occurring globally [80], where BA.5 descendent lineages continue to spread dominantly all over the world accounting for 81.2% of sequences. This is followed by BA.4 descendent lineages (BA.4.6) and BA.2 descendent lineages (BA.2.75) accounting for 8.1% and 2.9% of sequences, respectively. In addition, 7.8% sequences were unassigned but are suspected to be Omicron variant COVID-19 cases [80]. To control transmission of these variants, including Omicron, continuous monitoring by genomic surveillance is essential along with vaccination and pharmaceutical therapeutic interventions. The percentage of viral lineages by genomic surveillance of the proportions of circulating Omicron sub-variants in the United States, for the period of around 3 months from July to September 2022, is shown in Figure 2.

## 3. SARS-CoV-2 immunopathology

SARS-CoV-2 is an RNA virus which encodes a nucleocapsid phosphoprotein (N), membrane glycoprotein (M), envelope (E), spike glycoprotein (S) and nonstructural protein (nsp). The spike protein of the SARS-CoV-2 virus binds to the host cells as described above. The E and M proteins are involved in viral transcription, translation and assembly. The S glycoprotein tripolymer in a mature virion consists of two functional subunits S1 and S2 linked non-covalently. The S1 subunit consists of the N-terminal domain (NTD) and the receptor binding domain (RBD). Shang et al, were able to identify the functionally important epitopes in the SARS-CoV-2 RBD region by examining the crystal structure of RBD in complex with Angiotensin-converting enzyme 2 (ACE2) receptors, on the cell surface [82],[83]. The S2 subunit allows for the fusion of the SARS-CoV-2 virus S protein with the host cell membrane [82],[84],[85] during infection of a new cell, creating a fusion pore that releases the viral genome into the host cell cytoplasm.

SARS-CoV-2 possesses a polybasic cleavage site consisting of four amino acid residues at the intersection of subunits S1 and S2 of the S protein, rendering it susceptible to cleavage by furin and other proteases in the Golgi apparatus. The polybasic cleavage site is unique to the SARS-CoV-2 virus as it is flanked by the O-linked glucans addition to S673, T678 and S686 sites [4], but is absent from the related lineage *Betacoronaviruses*. Conformational changes in S protein aid in merging viral envelope and host cell membranes together, resulting in exposure of the internal S2′ site present on the S2 subunit of SARS-CoV-2. When RBD ACE2 interaction mediated endocytosis of SARS-CoV-2 virion occurs, the exposed S2′ site is cleaved by two major furin like proteases, the transmembrane protease, serine 2 (TMPRSS2) present on the host cell surface or by cathepsin L in the endosomal compartment. This cleavage of S2′ site in the S2 subunit initiates the fusion pore formation, ultimately allowing the viral genome to reach the host cell cytoplasm. Once the virion particle is inside the cytoplasm the biosynthesis of viral structural proteins and genomic RNA occurs in the endoplasmic reticulum (ER) and transported into the ER-Golgi intermediate compartment (ERGIC). The assembly of four viral proteins S, E, M and N occurs in the ERGIC. Once the virion particles are assembled, they are released into the ERGIC lumen and are secreted into the cytoplasm. The assembled virion particles escape to the extracellular space when the SARS-CoV-2 virus containing vesicles fuse with the plasma membrane. The detailed steps on the entry of the SARS-CoV-2 virus into the host cell leading to infection and inflammation were recently reviewed [86].

SARS-CoV-2 evolved in humans leading to specific point mutations in the S glycoprotein that promotes enhanced binding to the human ACE2 receptor. One example being the substitution of asparagine to tyrosine (N501Y) [87]. Many questions remain as to the future evolution of the S protein, which may influence the infectivity rate. Another immediate concern is the widespread expression of ACE2 in tissues, which could result in SARS-CoV-2 infection affecting multiple organs. Furthermore, SARS-CoV-2 can also interact with other molecules in addition to ACE2 to infect cells, such as CD147 and NRP1, which are also widely distributed in tissues [88].

## 4. Innate immune response

**Figure 3.**
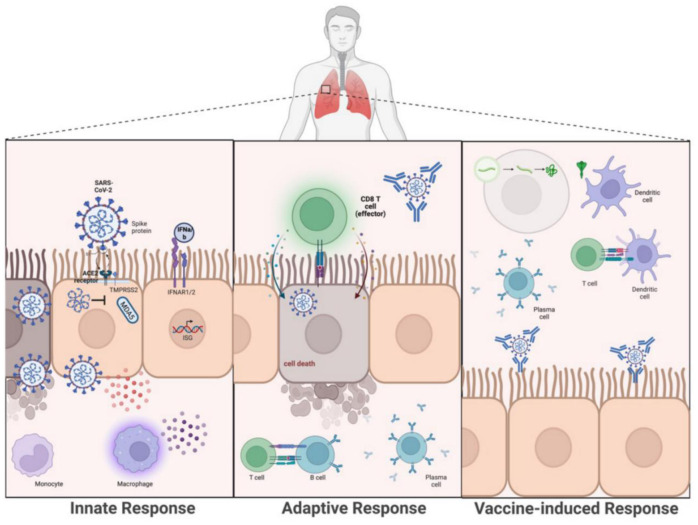
Immune response of natural infection with SARS-CoV-2 and vaccine-induced response against infection. The innate response to SARS-CoV-2 (left) is initiated by infection of the host cell expressing the ACE2 receptor by SARS-CoV-2 and recognition by pattern recognition receptors including the RIG-I-like receptor (RLR), and melanoma differentiation-associated gene-5 (MDA5). Successful activation of MDA5 leads to the downstream production of type I interferons (IFN) and cytokines. The produced cytokines and chemokines can recruit and activate inflammatory cells including monocytes and macrophages. IFNs bind to IFNAR on its own cell and neighboring cells. SARS-CoV-2 proteins can evade the immune response by inhibiting the action of MDA5. Adaptive responses (middle) are characterized by activated T helper cells (CD4+ Th cells) assisting B cell activation resulting in antibody production. Neutralizing antibodies mainly work to block viral entry into host cells. Activated CD8 T cells (cytotoxic CD8+ T cells) recognize viral antigens presented by the host major histocompatibility complex I (MHC I) molecules in infected cells and kill the infected cell by releasing cytotoxins granzyme and perforin. Vaccine-induced responses are targeted to specific proteins from the virus which, if effective, leads to protective adaptive immunity. In the example of mRNA vaccines (Right), administered mRNA is taken up by a cell and translated to produce the Spike protein. Antigen presenting cells such as dendritic cells, uptake the protein and process it to prime T cells.

The first encounter of the host with invading pathogens begins with an innate immune response. In the context of viral infections, the innate response is vital to dampening the rapid replication and spread of viruses. Physical barriers are the initial defense against pathogens and prevent the invasion of foreign particles and pathogens. These defenses consist of endothelia, skin, and mucous membranes which line the respiratory tract, gastrointestinal tract and urogenital tract. While the viscous mucus of the mucosal membranes acts as a physical barrier, in contrast to the other physical defenses, they also contain anti-microbial peptides and circulating IgA antibodies that can directly target pathogens. SARS-CoV-2 encounters the mucous membrane early into infection [89]. Inhaled particles initially contact the nasal mucosa lining the nasal epithelium. It was discovered early into the pandemic that SARS-CoV-2 can infect ciliated and mucus-secreting goblet cells that help compose the nasal epithelium and express ACE2 and TMPRSS2 [13]. Infection leads to viral replication and disrupts the structure of ciliated cells which help advance the infection. However, this stage of infection can lead to mild symptoms; since it is believed that at this point nasal epithelium can still recover given basal stem cells of the nasal epithelium are left uninfected. Given infection begins at the upper respiratory tract there have been calls for increasing research on mucosal immunity against SARS-CoV-2 [13],[85]. If the virus progresses towards the lower respiratory track this can lead to increased infection severity in the bronchi [84]. Once in the bronchi, the virus can target ciliated cells, club cells and mucus-secreting cells leading to epithelial injury [13]. With further progression to the alveoli, severe infection can ensue [13],[90]. Once viruses such as SARS-CoV-2 get past physical barriers and reach their target cells, innate immune responses are triggered [90],[91]. Multiple pattern recognition receptors (PRRs) within and on the surface of cells can recognize damage associated molecular patterns (DAMPs) and pattern associated molecular patterns (PAMPs); this includes endosomal toll-like receptors (TLRs) 3 and 7 and intracellular retinoic acid-inducible gene-I (RIG-I) receptors (RLRs), and melanoma differentiation-associated protein 5 (MDA5) [92],[93]. Activation of PRRs induces production of cytokines, chemokines and type I and III interferons. Chemokines aid in recruiting innate cells to the site of infection, including neutrophils, monocytes, natural killer cells, macrophages and dendritic cells (DCs) (Figure 3). Interferons act in both autocrine and paracrine signaling [94]. They bind their respective receptors on nearby cells with the resulting signaling cascade leading to broad induction of interferon stimulated genes (ISGs). Hundreds of ISGs have been identified and many have been discovered to possess antiviral functions. An *in vitro* experiment was conducted to broadly assess ISGs capable of restricting SARS-CoV-2, and this study led to the identification of 65 ISGs that indirectly or directly inhibited SARS-CoV-2 activity [95]. While interferon responses are pertinent to restricting viral activity, coronaviruses, including SARS-CoV-2, have been implicated in antagonizing the antiviral responses to evade clearance.

The non-structural proteins, accessory proteins and structural proteins of SARS-CoV-2 can suppress various components of the interferon response. The 16 nonstructural proteins of SARS-CoV-2 (NSP1-16) have been implicated in blocking host gene expression, and phosphorylation of IRFs and STAT1 [96]. Accessory protein, ORF9b is reported to interact and negatively regulate melanoma differentiation-associated gene-5 (MDA5), TIR-domain-containing adapter-inducing interferon-β (TRIF) and TANK-binding kinase 1 (TBK1), amongst others [97]. Similarly, SARS-CoV-2 membrane protein (structural protein) can bind MDA5 and TBK1 to block activity [98]. The ability of SARS-CoV-2 to suppress interferon responses allows for delayed clearance and uncontested replication. The rapid viral replication is believed to be responsible for the robust pro-inflammatory response exhibited by COVID-19 patients.

Early into the pandemic, those diagnosed with COVID-19 were found to display significantly elevated proinflammatory responses, including increased levels of IL-6, TNF, IL-2, IL-1β, IP-10, IFNγ, MCP1 and MIP1α [5],[99]. This notable robust cytokine response has largely become a hallmark of COVID-19. Studies have been able to identify the correlation between IL-6 levels and COVID-19 diagnosis as well as IL-6 and IL-8 levels with both COVID-19 diagnosis and severity [100]. Given the large importance of these cytokines, there has been a large emphasis on understanding the trigger for this robust response. Virus-induced pyroptosis of infected cells has been connected to initiating the high inflammatory response, with a large release of IL-1β. Also, pyroptosis leads to the release of PAMPs and DAMPs that can activate macrophages and monocytes, which produce cytokines and chemokines to recruit other cells to the site of infection such as T cells, triggering the robust cascade of inflammatory responses [101],[102]. The large influx of monocytes, macrophages and T cells is believed to drive lung inflammation and explain the increased neutrophil to lymphocyte ratios and lymphopenia exhibited in severe COVID-19 cases [103],[104].

## 5. Adaptive immune response

Both humoral and cellular immunity, including antigen-specific B and T cells, are key players in the adaptive immune response, which are essential for eliminating viral pathogens, including SARS-CoV-2 [105]–[107] (Figure 3). The B cell mediated humoral immune response, either vaccine-induced or induced by natural viral infection takes place in the circulation (extrafollicular (EF) immune response), in the germinal center of the lymph nodes and other peripheral lymphoid tissues. The EF immune response depends upon the antigen-specific interaction between T cells and B cells in the circulation which leads to the activation and rapid differentiation of B cells into short-lived plasma cells within a few days after viral infection which produce antibodies capable of neutralizing the virus [108],[109]. The antibodies produced by EF plasma cells are predominately IgM subtype, exhibiting fewer somatic hypermutations with low antigen affinity. However, depending upon the viral infection, EF plasma cells can be IgG or IgA isotype switched. Follicular helper (Tfh) T cells provide support to B cells in the germinal center by differentiating B lymphocytes into memory cells and long-lived plasma cells. Plasma cells generated in the germinal center secrete antigen-specific antibodies that are high in affinity and have undergone somatic hypermutation releasing isotype-switched antibodies [110]. These long-lived plasma cells and antigen-specific memory B cells reside in the bone marrow long after the patient is recovered naturally from the primary infection or after vaccination [111]. Naïve memory T cells and B cells in the bone marrow differentiate by clonal expansion and offer rapid protection against future viral re-infections. Memory B cells capable of producing neutralizing monoclonal antibodies depicting *in vitro* and *in vivo* activity have been isolated before from the SARS-CoV convalescent patient [112]. Antibodies developed from previous infections to coronaviruses conferring pre-existing immunity may cross-react and contribute to SARS-CoV-2-specific neutralizing antibodies including rapid EF IgG immune responses in primary SARS-CoV-2 infection [113],[114].

## 6. Humoral immunity

As a part of the humoral response, neutralizing antibodies provide protection against COVID-19 by targeting the RBD domain of the spike glycoproteins and nucleocapsid proteins of SARS-CoV-2 virus [115]–[117]. In a study reporting antibody responses in COVID-19 convalescent patients, it was observed that the plasma of COVID-19-convalescent patients contained low titers of neutralizing antibodies, but recurrent RBD-specific antibodies are present and capable of exhibiting potent anti-viral activity against the SARS-CoV-2 virus infection [118]. The level of neutralization activity in convalescent plasma samples however varies amongst COVID-19 patients [115],[119]. This aligns with the research studies performed at the start of the pandemic, generating mixed results in efficacy using COVID-19 convalescent plasma, when it was authorized as an emergency use therapy for patients experiencing severe or life threatening COVID-19 disease [120]. In a study investigating the levels of IgM and IgG immune responses in patients with mild and severe COVID-19 disease, it was observed that IgM antibody titers were detected from day 4 of the post symptom onset (PSO), reaching its peak around day 20 and subsequently reduced by week 4 PSO [121] in both mild and severe cases. Spike glycoprotein specific anti-SARS-CoV-2 IgG antibodies were detected from day 7 [115], reaching its peak around day 25 and existed in the serum samples at high levels after 4 weeks of COVID-19 infection depicting seroconversion. In patients with severe cases of COVID-19, both IgM and IgG antibodies showed vigorous responses [121],[122]. In a study aimed to understand the development of memory immune responses post COVID-19 infection, the authors measured the circulating SARS-CoV-2 specific memory B cells, CD8+ T cells and CD4+ T cells for 8 months from a cohort of 188 COVID-19 patients. It was observed that SARS-CoV-2 infection exhibited a high degree of heterogeneity within all the three arms of the adaptive immune response, including memory B cells, CD8+ T cells and CD4+ T cells amongst more than 90% of COVID-19 patients [123]. In a comprehensive study analyzing disease trajectory and immune responses generated amongst severe and recovered COVID-19 patients, it was observed that in severe cases of COVID-19, there is abundant oligoclonal B cell expansion generating antibodies with diverse CDR3 sequences [124]. The study confirmed development of a stronger humoral response against SARS-CoV-2 virus infection in severe COVID-19 patients eliciting higher viral load [125] compared to mild or recovered COVID-19 individuals [124]. Mucosal IgA antibodies, secreted in the monomeric or dimeric form in the respiratory tract, also contribute to eliminating or resisting SARS-CoV-2 transmission through the airway [89]. Neutralizing IgA antibodies secreted by plasmablasts are detected in saliva and nasal fluids PSO peaking around the third week of COVID-19 disease and last as long as two months PSO [89],[126]. As the levels of serum neutralizing antibodies induced post SARS-CoV-2 virus infection or vaccination start declining, there is a higher risk of reduced protection and re-infection from either the original Wuhan SARS-CoV-2 virus infection, VOCs or VOIs. Therefore, vaccine booster shots stimulating broader potent neutralizing antibodies should be developed to elicit B cell activation and humoral immune response in humans.

## 7. T cell mediated cellular immunity

Several recent studies have demonstrated the importance of CD4+ T cells [127], IFN-γ [128] and CD8+ T cells [129] on host immunity against SARS-CoV-2 (Figure 3) [130]–[132]. The observation that virally exposed individuals (PCR-negative and seronegative) have higher levels of SARS-CoV-2-specific T cells [133],[134] serves as supporting evidence that SARS-CoV-2-specific T cells mediate viral protection. The type of protective SARS-CoV-2-specific T cells is also an area of high interest [135]–[137]. Although both symptomatic and asymptomatic individuals possess SARS-CoV-2-specific T cells [133],[134], functional T helper 1 (Th1) cells, that secrete IFN-g and IL-2, are enhanced in asymptomatic individuals compared to symptomatic individuals[138]. Importantly, T cell responses can be modulated by SARS-CoV-2 through the inhibition of epithelial cell survival through the hypoxia inducible factor 1-alpha (HIF-1a)/glycolysis-dependent axis [139]. This may explain the reduced functionality of T cells and higher viral loads in patients with metabolic dysregulation [140].

The phenotype of the T cells associated with SARS-CoV-2 immune response is attracting more interest and could have wide implications for vaccine development. The CD4+ T cell population phenotypes in convalescent COVID-19 patients are predominantly central memory phenotype (CD45RO+, CCR7+), followed by an effector memory phenotype (CD45RO+, CCR7-). This suggests the persistence of antigen exposure and prolonged response with severity of infection being a determining factor [141]. In SARS-CoV-2 infection, when T cells are activated, CD4+ T cell (CD38+, HLA-DR+) phenotypes increase in infected individuals [142]. Additionally, the observed activated CD4+ T cell phenotype is mostly characterized by higher secretion of IL-2 [143]. Moreover, CD4+ T cell responses show balanced production of IL-10 and inflammatory cytokines in asymptomatic infection suspected to provide anti-viral responses without associated pathology, while in symptomatic COVID-19 patients disease response is more polarized [138],[144]. Examining the immune response in individuals who have experienced ‘long COVID’ and have cognitive impairment, such as ‘brain fog’ and difficulty concentrating, as well as depression, altered mood and other physiological symptoms, including fatigue, is crucial because these cases indicate incomplete recovery from COVID [145]. A recent study looked at long-lasting immune responses in convalescent patients with ‘long COVID’. Immune responses were assessed at 3 and 6 months after SARS-CoV-2 infection in each cohort [145],[146]. In this study, recovery from severe COVID-19 led to increases in proinflammatory cytokine responses from CD4+ and CD8+ T cells including TNFa, IL-2, IFN-g and CD107a at 3 months, followed by a significant decrease in these responses at the long-term timepoint (6 months), indicating a shift to an immunologically exhausted state of the CD4+ and CD8+ T cells [146]. Unresolved inflammation, shown by CD8+ T cells production of granzyme B and IFN-g was also observed in the severe COVID convalescent patients [146].

Importantly, CD8+ cytotoxic effector T cells (CTLs) that predominantly eliminate intracellular microbes by releasing cytotoxic factors, are a key player in the SARS-CoV-2 infection [136],[147],[148]. In acute COVID-19 infections, SARS-CoV-2-specific CTLs gain potent cytotoxic effector functions by releasing high levels of IFN-γ, granzyme B, perforin and CD107a molecules that are present in the cytotoxic granules [124],[135],[149],[150]. Asymptomatic and recovered COVID-19 patients show potent clonal expansion of CD8+ CTL subtypes [124].

Lung CD8+ resident memory T cells (Trm) play a crucial role in providing protection against respiratory mucosal infections caused by viruses[151]–[153]. This has prompted vaccine designers to focus on stimulating this specific T cell population. The development of lung Trm from effector T cells is dependent on the local recognition of antigens and the activation of growth factors such as TGF-β and interleukin (IL)-15 [154]. Mucosal vaccines can be the optimal tool for eliciting respiratory tract Trm cells immune responses since parenterally delivered vaccines are less likely to induce lung Trm cells [154].

Dendritic cells (DCs) are the immune response's most potent antigen-presenting cells (APCs) that can present SARS-CoV-2 peptides and proteins to T cells [155]. T cells recognize simple short peptides (8–15 amino acids) presented by DCs in the context of human leukocyte antigen (HLA) class I or class II molecules. Interestingly, the proportion of DCs in patients with mild COVID-19 was not significantly changed. However, in patients with severe COVID-19 , the number of DCs was lower than in patients with mild COVID-19 [156]. Interstitial lung DCs express intermediate levels of ACE-2. This suggests that these interstitial lung DCs can be infected by SARS-CoV-2 [157]. Additionally, it was shown that CD147, a transmembrane glycoprotein, can act as a receptor allowing SARS-CoV-2 entry in DCs [158].

T cells are activated excessively in severe cases of COVID-19. In studies examining host immune correlates of protection, it was observed that there is a significant increase of HLA-DR expression and IFN-γ production in CD4+ T cells [107],[159]. In contrast, CD8+ T cells population decrease was observed in COVID-19 recovered patients [160],[161]. Another important subset of T cells, which negatively regulate the immune response, are regulatory T cells (T_reg_) cells. The T_reg_ cell population was observed to be reduced in severe cases of COVID-19 [162]. This marked decrease in T_reg_ cells explains the state of excessive activation of T cells [162]. Furthermore, the dysregulation of the Treg/Th17 ratio is attributed with increased COVID-19 disease severity and aggravated pulmonary inflammation [163]. Th17 phenotype, transcription factor GATA's response to IL-17, IL-22 dependent T cell subset, is also associated with elevated inflammation in lung interstitium. The marked increase in COVID-19 disease severity is a result of neutrophil migration and G-CSF, TNF-a, IL-1b and IL-6 activation [164].

It was observed, during SARS-CoV-2 infection, that antibody responses are the main players of protection due to the presence of virus-specific neutralizing antibodies within the airways. However, high antibody levels have been associated with increased inflammation [165]. Indeed, CD4+ Th2 cell response is associated with the increased severity of SARS-CoV-2 infection. COVID-19 patients showing ARDS have a disproportionate Th1:Th2 cell ratio that triggers an excessive inflammatory response in the lungs. [166],[167]. In general SARS-CoV2 induces Th2, Th1/Th17 cells in COVID-19 patients, but the subsequent pathogenesis and multi-organ dysfunction are the consequence of the specific cytokines released during the SARS-CoV2 infection [167].

An important subtype of T cells associated with producing long-lasting, high-affinity antibody response is the T follicular helper (Tfh) cells [168]. Tfh cells are developed from activation of T cells in the extrafollicular region of the lymphoid tissue [168],[169]. Vaccinated individuals show that activation of Tfh in the extrafollicular region persists over a longer period [170],[171], leading to development of high titer neutralizing antibody responses. Overall, robust T cell immune responses are crucial for the generation of high-titer neutralizing antibody responses following COVID-19 infection or vaccination.

## 8. COVID-19 vaccine development and efficacy

**Figure 4.**
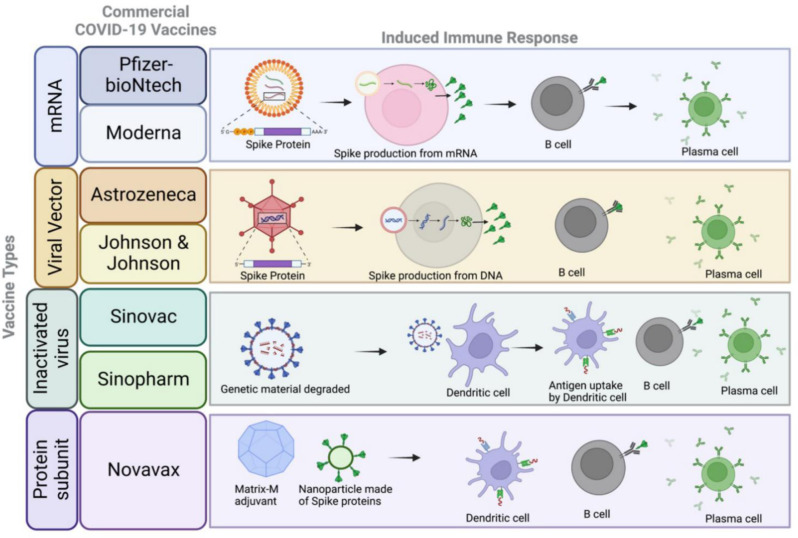
The immune responses driven by different COVID-19 vaccine platforms. The mRNA platform by Moderna and Pfizer administer mRNA encoded for spike protein, for cellular uptake and translation of the mRNA to protein. The produced spike protein largely leads to activation of B cells to plasma cells for neutralizing antibody production. The viral vector platform used by AstraZeneca and Johnson & Johnson engineer adenovirus vectors carrying genomic material for the spike protein that transfects cells inserting the DNA for production of the protein. Similarly, this leads to B cell and plasma cell production. Inactivated virus platforms used by Sinovac and Sinopharm chemically degrade the genomic material of SARS-CoV-2. Once administered the inactivated virus acting as an immunogen can be taken by antigen presenting cells such as dendritic cells that can lead to the downstream activation of T cells and B cells, and the production of antibodies. The protein subunit vaccine by Novavax includes the nanoparticle composed of spike proteins and the saponin-based Matrix M adjuvant that can be detected and presented by the APC, dendritic cell, for T cell and B cell activation.

The new SARS-CoV-2 virus (with a worldwide population of immunologically naïve individuals) and the severity of COVID-19, led to a rapid, global response to developing an effective vaccine. Development of vaccines on average span multiple years, if not decades. In the case of COVID-19, it took less than a year's time to get vaccines developed for administration. By early 2021, nearly a year since the start of the pandemic, there were multiple vaccines in the pipeline for human distribution, including leading mRNA vaccines by Pfizer-BioNTech and Moderna, and adenovirus-based vaccines by Johnson & Johnson and AstraZeneca (Figure 4). The rapid development of the leading vaccines is in part due to the years of previous research on new vaccine technologies and Coronaviruses, as well as the expansive global effort in allocating resources towards vaccine development [172].

For decades now mRNA research has continuously expanded in part due to the potential of its immunotherapy properties. For mRNA vaccines to be successfully delivered, they must avoid degradation once administered, be internalized by host cells, and then reach the cytoplasm for translation and protein production [173]. The creation of lipid nanoparticles was crucial for overcoming these barriers for mRNA delivery. Encapsulation of mRNA in lipid nanoparticles protects mRNA from degradation and improves its stability [155],[156],[173],[174]. With safe delivery, the mRNA-lipid formation can be internalized by host cells through micropinocytosis, and clathrin-mediated endocytosis, amongst others. Then the mRNA is transiently translated within host cells into the targeted protein. At this stage the protein can be processed through multiple routes to develop an adaptive immune response. The protein can be processed by the cell for MHC class I presentation to CD8^+^ T cells, MHC class II presentation to activate CD4^+^ T cells, or packaged in exosomes allowing for B cell recognition [175],[176]. This leads to robust and persistent adaptive responses, with up to eight weeks of sustained mRNA observed within the germinal centers [176],[177]. In the case of the recent COVID-19 vaccines by Moderna and Pfizer, the injected mRNA is coded to translate the SARS-CoV-2 spike protein. Intramuscular injection of the mRNA leads to the mRNA being taken up by APCs in the muscle which then migrate to the draining lymph nodes where they can interact with B and T cells. This leads to robust activation of adaptive immunity, including the production of neutralizing antibodies, targeted to the spike protein. Upon infection with SARS-CoV-2, the spike-specific antibodies in addition to vaccine-specific cell-mediated immune responses, are primed and available to provide vaccine-mediated protection. The novel mRNA technology is revolutionary in that it utilizes the body's own cells to produce the targeted protein, whereas for many years vaccine platforms consisted only of viral vectors, inactivated pathogens, and protein subunits.

Viral vector vaccines are primarily derived using adenoviruses, non-enveloped double stranded DNA viruses, engineered to express target proteins and are unable to replicate inside the hosts to avoid pathogenic effects. The vaccine by Johnson & Johnson uses a viral vector based on the human adenovirus 26 and encodes the spike protein [178]. The AstraZeneca vaccine also uses an adenoviral vector, the chimpanzee Ad25 vector expressing the spike protein [179]. Both vaccines rely on the virus to enter cells via clathrin-mediated endocytosis. Once inside, the viral capsid is dismantled. The replication deficient viral DNA containing SARS-CoV-2 genes of interest enters the nucleus, where the DNA is transcribed, leading to the production of immunogenic SARS-CoV-2 proteins in the cytoplasm [180],[181]. For the vector vaccines containing the code for spike protein, cells ‘infected’ with the adenovirus vector will produce the spike protein leading to production of neutralizing antibodies against SARS-CoV-2 spike and induction of a pro-inflammatory Th1 response [182]. Inactivated vaccines contain the pathogen of interest, chemically inactivated so it can be safely administered and able to induce a broad cellular and humoral immune response [183]. Compared to most other vaccine platforms that target a specific protein, inactivated vaccines use the entire pathogen as an immunogen to widen the repertoire of antibodies and T cells. However, since the pathogen is non-replicative, a higher concentration is usually needed. Vaccines by Sinovac and Sinopharm utilize SARS-CoV-2 propagated in Vero cells, inactivated by beta-propiolactone and adjuvanted with alum; however, they use different early strains of SARS-CoV-2 [184]. Protein subunit vaccines have and continue to be used; examples including the hepatitis B and whooping cough vaccines. In contrast to inactivated vaccines, the protein subunit platform uses a component of a pathogen to induce an immune response. Viral vector, mRNA and protein subunit vaccines all utilize the Spike protein as the vaccine immunogen; however, the viral vector and mRNA vaccines aim to produce spike protein as their immunogen, whereas it is administered directly in protein subunit vaccines. The Novavax protein subunit vaccine produced full length, prefusion spike protein using the insect Sf9, baculovirus expression system [185]. The spike proteins are assembled into nanoparticles and adjuvanted with Matrix-M, a saponin-based adjuvant, to bolster immune responses.

The urgent need to deploy vaccines and the rapid administration meant the long-term effectiveness of the vaccines was still being evaluated while active in the population. There are different criteria to consider when evaluating vaccine efficacy, such as the ability to prevent severe disease, hospitalization, symptoms, or infection. Vaccination against SARS-CoV-2 virus does not block infection but generates an effective immune response that reduces disease progression and disease severity [186]. During phase III trials most of the approved vaccines showed high efficacy against severe disease and symptoms (Table 3). However, it was still unknown if the vaccines could inhibit transmission and given that at-risk groups and children were still ineligible for vaccination, it was a concern if vaccinated persons could transmit the virus (if infected) to the vulnerable populations. Community and household studies did find that rate of transmission was lower amongst vaccinated groups compared to unvaccinated [187],[188]. However, the constant emergence of SARS-CoV-2 variants posed an issue for long-term efficacy and introduced many breakthrough infections amongst the vaccinated population [189]. Booster shots containing updated spike proteins of the emerging variants were initiated to combat the new wave of infections.

**Table 3.** Types of commercial vaccines against COVID-19 and their efficacy in humans.

Vaccine	Platform	Location of origin	WHO emergency use date	Administration schedule	Efficacy*
Pfizer-BioNTech (BNT162b2)	mRNA	United States Germany	31 Dec 2020	2 doses, 21 days apart	95.6% (<65) 94.7% (≥65) [190]
Moderna (mRNA-1273)	mRNA	United States	30 Apr 2021	2 doses, 28 days apart	95.6% (<65) 86.4% (≥65) [191]
Johnson & Johnson-Jassen (Ad26.COV.2.S)	Viral vector	Belgium	12 Mar 2021	Single dose	56.6 (<59) 55% (≥60) [192]
AstraZeneca (ChAdOx1 nCoV-19)	Viral vector	England	15 Feb 2021	2 doses, 28 days apart	72.8% (<65) 83.5% (≥65) [193]
Novavax (NVX-CoV2373)	Protein subunit	United States	20 Dec 2021	2 doses, 21 days apart	91.5% (<64) [185]
Sinopharm (Covilo)	Inactivated	China	07 May 2021	2 doses, 21 days apart	99.98% (<60 years) 99.12 (≥60 years) [194] (REF)
Sinovac (CoronaVac)	Inactivated	China	01 Jun 2022	2 doses, 14 days apart	83.5% (<59) [195]

Furthermore, the need for long-lasting, accessible and effective vaccines has pushed the vaccine field into advancing vaccine technology. An emerging vaccine by HDT Bio utilizes the virus-derived replicon RNA (repRNA) vaccine platform. The vaccine is formulated with lipid inorganic nanoparticles (LIONs) and self-amplifying RNA encoded for the spike protein allowing for robust and ongoing antigen production within a cell [196]. While there are still improvements that need to be made in accessibility, testing different routes of immunizations and providing stable protection, the advancing vaccine technology and the swift response to producing effective vaccines illustrates the continued growth of vaccinology. In addition, continued effort to understand the mechanism of efficacy and safety for each of the vaccine platforms is of the utmost importance.

## 9. Qualification and validation of endpoint assay methodologies to measure immune responses

Effective vaccines against SARS-CoV-2 should induce innate and adaptive immune responses. Prior to Emergency Use Authorization (EUA) SARS-CoV-2 vaccines were evaluated for their safety, immunogenicity and efficacy. Clinical trials rely on endpoint assays to evaluate the immunogenicity of vaccines in the pipeline. These endpoint assays need to be validated to obtain reliable and reproducible results for a specific methodology. Few validation parameters that are measured during qualification of an endpoint assay include robustness, dilutional linearity and precision [197],[198]. To measure effective immune responses generated against SARS-CoV-2 infection, Serological or endpoint titer evaluation of IgA, IgM and IgG ELISAs as well as pseudo neutralization and live viral neutralization assays were developed to test the ability of candidate vaccines to induce protective immune responses [199]. To evaluate immunogenicity, vaccines in the clinical pipeline were evaluated using qualified or validated endpoint assays for their ability to induce neutralizing titers of antibodies from serum samples of COVID-19 vaccine recipients. Additional assays, that were qualified or validated by our group and others include a plaque reduction neutralization test (PRNT) assay and a pseudo virus neutralization assay which evaluates functional antibody titers capable of inhibiting the entry and replication of a lentivirus containing the Spike antigen of SARS-CoV-2 [199]. These assays were validated for precision, assay repeatability, intraday variability, interday variability, linearity and specificity by evaluating the magnitude and protective immune response against SARS-CoV-2. These qualified and/or validated serological assays have become crucial for the evaluation of COVID-19 vaccine efficacy in the clinical pipeline.

## 10. Conclusion

SARS-CoV-2 variants may act differently or can impact how quickly the virus spreads. Depending upon the unique mutations occurring in the genome of the variants, the severity of illness that COVID-19 causes or the effectiveness of vaccines and treatments against SARS-CoV-2 infections may change, and like the influenza vaccine, a new vaccine may be required annually. Although updated vaccines and new treatment methods are constantly emerging in many countries, all efforts should be made to ensure that the distribution is equitable to prevent the susceptibility of large populations of people to infection with emerging SARS-CoV-2 mutants. While science and technology have made large strides towards the development of vaccines and therapies against SARS-CoV-2, there is still much that is incompletely understood. Therefore, urgency is still required to better understand the viral/host pathogenicity if new and improved therapies, antivirals and vaccines continue to be developed and brought to market.
